# Ideopathic Tetanus

**Published:** 1883-01

**Authors:** G. P. Delisser

**Affiliations:** New York City


### III.—IDIOPATHIC TETANUS.

REPORTED BY G. P. DELISSER, V.S.

I was called August 29th, 1882, to see a roan horse suffering from idiopathic tetanus, owned by a wholesale butcher and raised in his business The animal had been under the care of a well known and reputable veterinarian of this city from August 23d, who had diagnosticated the disease early and put him under treatment, such as blisters over the spinal column, free cathartics, but gave an unfavorable prognosis and was consequently discharged. In his opinion, however, I was disposed to concur, but there was some hope in a line of treatment which I had adopted in a similar case.

The leading points in the history of this case are, that August 22d the animal was, to appearances, in perfect health. On that day, he was driven to Brooklyn with a load and returned late in the evening during a heavy rain; was stabled that night and fed as usual.

The morning of August 23d, the groom found that his supper was untouched and the horse sick, with the following symptoms: stiff in moving, and labored breathing.

The veterinarian was at once summoned and the above treatment advised and carried out, but with a negative result.

His appearances at the time of my first visit were extreme nervousness, quivering in almost all of the superficial muscles, breathing rapid, temperature 100° F., nostrils expanded, jaws tightly closed, neck stiff and thrown upwards, slight salivation. There was also great difficulty in moving, even so much that I expected he would fall; bowels constipated, urine very scanty.

*Treatment.*—Cleared the rectum with my hand, and with difficulty removed several masses of hardened fœces. Gave enema of Oleum Lini and ʒii. of Extract Belladonnæ. Had the back and loins bathed with warm water and the blisters dressed with linseed oil and lime water, equal parts of each. Administered gr. vi. of morphine in solution by the mouth every hour until the spasmodic twitching of the muscles was checked, which was accomplished in about twelve hours. Gave enemas of boiled Indian and oat meal for nourishment, about a pint of each at a time in water, twice daily.

August 30.—No marked change. Muscular spasms less marked. Morphine administered every six hours. Same treatment continued.

August 31st.—Muscular contractions diminishing, breathing less hurried, jaws less ridged, in fact they could be opened with comparative ease and the horse swallowed a ball. The ball administered in this way contained Ex. Bel.

ladonnæ ℥ss., Ex. Hyoscyamus ℥ss., Oleum Tiglei M. xx. Morphine stopped, rectal alimentation continued.

September 1st.—Improvement more marked. Balls, containing Ex. Bella donnæ ℥ss. each, were given, and the Hyoscyamus added to the enemas.

Applied several times a day along the vertebral column and over the jaw the linement:

℞

| | |
|---|---|
| Chloroform, | ℥ii. |
| Ext. Belladonnœ, Fld., | ʒii. |
| Oleum Terebinthe, | ℥ii. |
| Oleum Olive, | ℥v. |
| Mx. Sig. Ex. use. | |

September 2d.—Marked improvement, bowels moved freely, urine more abundant, respirations quite free, incisor teeth could be separated about one inch, washed the tenacious saliva from the mouth with a warm salt solution. Gave the following laxitive ball.

℞

Pulv. Aloes, ℥ss.
Ext. Nux. Vomic, et.
Ext. Belladonna, et.
Ext. Hyoscyam aa., ʒii.
Mx. Sig. one ball.

Use of linement continued.

September 3d.—Could open mouth and eat a scalded mash of oats and meal. All previous treatment suspended and small doses of Opium and Bromide of Potassium added to his meals three times daily.

Sept. 4th.—Steadily improving, but has a little difficulty in drinking, bowels regular, micturation frequent, and urine highly colored, breathing nearly normal, muscles a little stiff. Opium suspended and small quantities of the iodide of potass given.

September 7th.—Could lie down and rise without assistance, all treatment stopped.

September 9th.—Quite well.

New York City.

